# Knowledge of vitamin D and practices of vitamin D supplementation in a Scottish adult population: A cross-sectional study

**DOI:** 10.1177/02601060241238824

**Published:** 2024-03-18

**Authors:** Suzanne M.M. Zaremba, Karen Conduit-Turner

**Affiliations:** 1 Division of Population Health & Genomics, Ninewells Hospital & Medical School, 3042 University of Dundee, Dundee, UK; 2 Public Health, 1251 NHS Tayside, Dundee, UK

**Keywords:** Vitamin D, supplementation, knowledge, awareness, Scotland

## Abstract

**Background:** Vitamin D supplementation practices (dose and frequency) are relatively unknown in the Scottish population, with no recent up-to-date data available. Reassessing current knowledge, practices, and awareness of vitamin D supplementation following a national health campaign in 2020 by Food Standards Scotland on vitamin D is warranted. **Aim:** This article aims to present the knowledge and awareness of vitamin D, and current vitamin D supplementation practices in adults living in Scotland. **Methods:** A cross-sectional study was performed between June and July 2022 using an online survey adapted from previous work on assessing knowledge of vitamin D in adults. Participants aged 18+, living in Scotland for ≥6 months were eligible to participate. Scores for knowledge were calculated as a percentage. Univariate associations between demographic and supplement use were established by *χ*^2^-test and logistic regression performed to predict factors associated with daily vitamin D intake. **Results:** Four hundred and three participants (72.7% female), mean age 36.4 (±14.2 years), completed the study. Awareness of vitamin D was very high (99.5%) but the mean overall knowledge score was poor (31.4 ± 15.3%), with those with a university degree more likely to have knowledge scores at/above the mean compared with those with lower levels of education, *χ*^2^(1, N = 393) 10.7, p = 0.001, odds ratio (OR) = 2.1 (95% confidence interval (CI) 1.7–2.7). Finally, 64.3% took vitamin D supplements, of which 37.5% took them daily during winter months, with only 7.4% taking the recommended daily dose. **Conclusion:** The current study highlights the need to improve both knowledge of vitamin D and practices of vitamin D supplementation during the autumn and winter months in Scotland.

## Introduction

Vitamin D (cholecalciferol) is a fat-soluble vitamin that is synthesised primarily in the skin via activation of 7-dehydrocholesterol by ultraviolet (UVB type) sunlight. Vitamin D can be acquired through dietary sources, including oily fish, eggs and dairy, however these are limited, and meeting requirements can be challenging through dietary means alone (British Dietetic Association, 2019). Therefore, supplementation is the focus for most policies aiming to improve vitamin D levels in the UK population. Vitamin D deficiency, defined as plasma 25-hydroxivitamin-D < 25 nmol/L (Scientific Advisory Committee on Nutrition, 2016), is problematic in Scotland with nearly half of those in the most deprived areas (47%) with insufficient vitamin D to protect their musculoskeletal health, compared with a quarter of those in the least deprived areas (Purdon et al., 2013). Prevalence of vitamin D deficiency in Scotland has been reported to be higher than other UK nations (Hyppönen and Power, 2007). Due to the high northern latitude of Scotland (55° to 59° North) and cloudy weather, conditions do not often favour vitamin D synthesis via UVB mechanisms (Rhodes et al., 2010). In addition, spending time indoors, obesity, high alcohol intake, and poor diet adversely affect vitamin D status.

Not only is Scottish data lacking with regard to up-to-date data on the prevalence of vitamin D deficiency, there is limited understanding of vitamin D supplementation practices in adults living in Scotland. The Scottish Government recommendation, in line with SACN guidance, is for adults to consider taking 10 micrograms (µg) of vitamin D daily between October to March (Scientific Advisory Committee on Nutrition, 2016; Scottish Government, 2022a). This advice relates to public health advice for vitamin D supplementation in the general adult population. This advice does not apply to specific medical cases or elderly patients where high loading doses to correct for deficiency status may be relevant. This research is focussed on the general adult population only, in whom 10µg is an appropriate daily dose with very low risk of toxicity.

UK survey data published in 2021 by the British Nutrition Foundation showed that 26% took vitamin D supplements all year round, with 8% of British adults taking vitamin D supplements most of the year and 8% taking them during autumn and winter (British Nutrition Foundation, 2021). Over a third of people (39%) reported never taking vitamin D supplements. However, the reported figures did not detail whether these were the recommended dose (10 µg) or taken daily. A cross-sectional survey by O’Connor et al. (2018) showed similar levels of supplementation within the UK population, 43.5% of individuals took vitamin D regularly but again data did not report if supplementation practices were daily during autumn-winter or the percentage of those taking the recommended dose.

According to Scottish Health Survey (2018) data 14% of Scottish men and 23% of Scottish women take vitamin D supplements, however dose and frequency were not reported. In 2020 Food Standards Scotland (FSS) conducted a survey to assess public adherence to the vitamin D recommendation. They reported that 58% of participants (n = 1465) were not taking vitamin D supplements, to which the main reason was not being aware of the guidance or what the recommended dose is (Food Standards Scotland, 2020). Subsequently, in 2021–2022 Food Standards Scotland ran a campaign which publicised the Scottish Government recommendation for vitamin D supplementation alongside health information regarding vitamin D. The campaign was run on TV, social media/online, and pop-up field marketing across Scotland (Food Standards Scotland, 2022a). With the UK Government's recent call for evidence and research on vitamin D (UK Government, 2022) it was timely to investigate whether knowledge of vitamin D has improved following the national FSS campaign, assess what barriers to supplementation still exist and identify what the current supplementation practices are in the Scottish adult population. To the researchers’ knowledge, this is the first study to quantitatively investigate vitamin D knowledge and supplementation practices, including amount and frequency, in adults living in Scotland.

## Materials and methods

### Study design, population and timeline

An observational, cross-sectional study was carried out from 15 June to 6 July 2022. Eligibility criteria included male and female adults (≥ 18 years). In order to assess knowledge of vitamin D and practices of vitamin D supplementation following the winter Food Standards Scotland campaign (run nationally across Scotland from October 2021 to Febraury 2022; Food Standards Scotland, 2021) eligible adults were required to have been resident in Scotland for a minimum of 6 months or longer for potential exposure to the campaign.

### Questionnaire

Knowledge and awareness of vitamin D and vitamin D supplementation practice in Scotland were assessed using an online questionnaire, administered via Jisc (Jisc, 2022). Potential participants in this study were invited to participate via social media platforms (Twitter, Facebook, LinkedIn and Instagram), which included a direct link to the questionnaire, study information and consent. Participants were also recruited via University of Dundee newsletters, University of Dundee student societies and Blackboard announcements for staff and students.

The questionnaire was developed using previous studies investigating vitamin D knowledge (Boland et al., 2015; O’Connor et al., 2018), and awareness of vitamin D (Food Standards Scotland, 2021). Questions on vitamin D practices were adapted from a previous national survey on vitamin D (Food Standards Scotland, 2021). Questions from the Scottish Census were used to gather demographic data (Scottish Government, 2022b). Postcodes were used to determine Scottish Index of Multiple Deprivation (SIMD), using the SIMD 2020 postcode lookup tool (Scottish Government, 2020). The questionnaire consisted of 33 questions, containing five sections; demographics, vitamin D awareness, knowledge of vitamin D, vitamin D practices and vitamin D supplements (Appendix 1). To ensure face validity, the researchers reviewed each question in the survey, and knowledgeable individuals (n = 6) in the field of vitamin D conducted an expert review to assess the appropriateness of the survey questions and response options. Additionally, piloting of the questionnaire was undertaken in a sample of University of Dundee staff and students (n = 12) for content validity.

Knowledge scoring was performed as per Boland et al. (2015) with each correct answer worth one point, producing a maximum score of seven points. Questions that required only one response were marked as correct, and gained one point, or incorrect for zero points. For questions that included multiple correct responses, each correct response was worth an equal fraction of the overall question score. Questions related to effects and sources of vitamin D both had eight correct answers meaning each one selected was worth 1/8, for factors that decrease vitamin D each was worth 1/13, each correct month of the year was worth 1/6. Equally weighted fractions were deducted for incorrect answers. This penalty for guessing was implemented to prevent participants from scoring 100% on multiple response questions by selecting all possible responses to that question. For this reason, the response “I don’t know” was not penalised. The final knowledge score was recorded as a percentage. Scores were then stratified into less than average and better than average to perform univariate association analysis between categorical variables and knowledge scores. The mean knowledge score was 31.4 ± 15.3%.

For both the recommended daily dose of vitamin D knowledge question and the entry of actual daily dose taken by participants the data entered was inconsistent. Despite the survey question requesting that the participant enters the dose in µg, some participants entered in international units (IU) and identified it as such, however others did not specify a unit. In order to interpret whether the dose was correct or incorrect an assumption was made that doses >100 referred to IU and anything below that referred to µg, unless otherwise stated. The rationale being that most daily vitamin D supplements are ≤100 µg and this is the upper tolerable limit (European Food Safety Authority, 2016; Rizzoli, 2021).

### Statistical analysis

The Statistical Package for Social Science (IBM SPSS software; version 26, IBM, NY, USA) was used to analyse all data.

Descriptive statistics were used to present demographic data and to evaluate knowledge, awareness and practices of vitamin D supplementation. For data that was normally distributed, mean and standard deviations are reported, the median and interquartile range are reported for data that did not have a normal distribution. Univariate associations between categorical variables (age, gender, employment, SIMD, income, ethnicity, and nationality) and supplement use were established by χ^2^-test. To establish factors contributing to daily vitamin D supplement use logistic regression analysis was performed. Factors with a p-value < 0.1 were included in the regression model. Results are expressed from the logistic regression using odds ratios, 95% confidence intervals and a p value < 0.05 was considered statistically significant.

The Strengthening the Reporting of Observational Studies in Epidemiology (STROBE) guidelines for the presentation and writing of this manuscript were followed (Von Elm et al., 2007).

## Results

### Demographics

In total 403 participants completed the survey. The mean age was 36.4 (±14.2 years), with one datapoint excluded due to an invalid age entry. The participants were predominantly female (72.7%), of which 2.0% were pregnant or breastfeeding. Most participants were in full-time employment (43.4%), with 45.6% earning £700 per week/£36,400 per annum or above. Over a quarter of respondents were from the highest SIMD, quintile 5. Education level was high with 60.5% participants educated to degree level or above and 15.9% had obtained school completion qualification equivalent or above such as Scottish Highers/GCSEs. The median time living in Scotland was 22.3 years with a range from 0.5 to 77.5 years. In terms of ethnicity 83.4% identified as white and for nationality although Scottish was in the majority when comparing individual nationalities, it made up less than 50% overall. Participants’ demographics are summarised in Table 1.

**Table 1.** Demographic patterns of participants (n = 403).

Demographic	Group	n	Percent
Age	18–24	118	29.3
	25–34	86	21.3
	35–49	112	27.8
	50–64	71	17.6
	65+	15	3.7
	Missing	1	0.2
Gender	Male	100	24.8
	Female	293	72.7
	Prefer not to answer	2	0.5
	Other	6	1.5
	Missing	2	0.5
Pregnant/breastfeeding	Yes	8	2
	No	392	97.3
	Prefer not to answer	1	0.2
	Missing	2	0.5
Employment	Full-time employment	175	43.4
	Part-time employment	63	15.6
	Not currently employed	18	4.5
	Student	123	30.5
	Retired	18	4.5
	Prefer not to answer	2	0.5
	Missing	4	1
Income	Less than £100 pw OR less than £5200 pa	18	4.5
	£100–199 pw or £5200 to £10,399 pa	27	6.7
	£200–299 pw or £10,400 to £15,499 pa	28	6.9
	£300–399 pw or £15,500 to £20,799 pa	17	4.2
	£400–499 pw or £20,800 to £25,999 pa	19	4.7
	£500–699 pw or £26,000 to £36,399 pa	43	10.7
	£700–999 pw or £36,400 to £51,999 pa	61	15.1
	£1000–1499 pw or £52,000 to £77,999 pa	66	16.4
	£1500 pw or more to £78,000 or more pa	57	14.1
	Prefer not to answer	63	15.6
	Missing	4	1
SIMD quintile	1 (most deprived)	47	11.7
	2	73	18.1
	3	79	19.6
	4	96	23.8
	5 (least deprived)	104	25.8
Nationality	Scottish	162	40.2
	Non-Scottish	233	57.8
	Missing	8	2
Ethnicity	White	336	83.4
	Mixed or multiple ethnic groups	5	1.2
	Asian, Scottish Asian or British Asian	39	9.7
	African, Scottish African or British African	13	3.2
	Caribbean or Black	0	0
	Other ethnic group Arab, Scottish Arab or British Arab, Other	7	1.7
	Missing	3	0.7

pa: per annum; pw: per week; SIMD: Scottish Index of Multiple Deprivation.

### Awareness of vitamin D and food standards Scotland campaign

All participants (99.5%), apart from three (0.5%), had heard of vitamin D. The commonest source of vitamin D information was school/college/university (56.4%), followed by family (45.1%), or from a doctor or nurse (37.9%). Other sources included friends (29.4%), social media (28.9%), television (28.2%) and less frequent sources were books and newspapers. When asked if they were aware of the current recommendation to take a daily supplement in autumn and winter, 65.5% answered ‘yes’, with 31.5% (n = 127) then answering what they believed the recommended dose was. Only 8.2% of participants recalled seeing the FSS vitamin D campaign adverts, and 10% could not recall it. The commonest sources for those who had seen the campaign were social media 37.8% (n = 17), television 20.0% (n = 9), 15.6% poster/billboard (n = 7), 8.9% newspaper (n = 4) and 4.4% radio (n = 2). ‘Other’ responses (13.3%) included at work, in a store, health centre and the internet/website advertisement as the source.

### Vitamin D knowledge

The mean vitamin D knowledge score (±SD) was 31.4 (±15.3%) with 43.4% (n = 174) scoring above average. There was a statistically significant association between knowledge scores (stratified into < and > mean of 31.4%) and education level. Those who were educated to a degree level or above were more likely to have scored higher than average (OR = 2.1, 1.7–2.7), and those who were educated below university degree level were more likely to score less than average, *X*^2^ (1, N = 393)* *= 10.7, p = 0.002. There were no statistically significant associations between knowledge and any other demographic factors, including age, gender, income, SIMD, employment status, ethnicity, nationality, pregnancy status and length of residency in Scotland (p > 0.05). Participants’ knowledge of vitamin D is summarised in Table 2.

**Table 2.** Participants’ knowledge of vitamin D; questions used in vitamin D knowledge score calculation (n = 401).

Question topic		✓/ ×	N (% participants who selected answer)	
Benefits of vitamin D	Bone health	✓	325 (80.6)	
	Immune health	✓	283 (70.2)	
	Prevention of Rickets	✓	234 (58.1)	
	Vision health	×	81 (20.1)	
	Hair growth	×	99 (24.6)	
	Prevention of diabetes	✓	53 (13.2)	
	Cardiovascular health	✓	97 (24.1)	
	Cognitive health	✓	118 (29.3)	
	Cancer prevention	✓	75 (18.6)	
	Skin softness	×	41 (10.2)	
	Calcium absorption	✓	242 (60.0)	
	None of the above	×	1 (0.2)	
	I don’t know	-	30 (7.4)	
	Other	-	12 (3.0)	
Sources	Fruits	×	97 (24.1)	
	Vegetables	×	124 (30.8)	
	Fatty fish	✓	176 (43.7)	
	Vitamin D supplements	✓	330 (81.9)	
	Select cereals	✓	133 (33.0)	
	Milk/dairy	✓	138 (34.2)	
	Nuts	×	58 (14.4)	
	Cod liver	✓	133 (33.0)	
	Sun	✓	338 (83.9)	
	Eggs	✓	121 (30.0)	
	Mushrooms	✓	66 (16.4)	
	I don’t know	-	9 (2.2)	
Factors that decrease absorption	Skin pigment	✓	212 (52.6)	
	Pregnancy/lactation	✓	113 (28.0)	
	Dairy allergy	✓	92 (22.8)	
	Shade/ clouds	✓	235 (58.3)	
	Fatty diets	×	47 (11.7)	
	Pollution	✓	126 (31.3)	
	Time of day	✓	206 (51.1)	
	Sunscreen use	✓	155 (38.5)	
	Wind	×	9 (2.2)	
	Latitude	✓	141 (35.0)	
	Vegan diet	✓	124 (30.8)	
	Smoking	×	74 (18.4)	
	Season	✓	210 (52.1)	
	Vegetarian diets	✓	82 (20.3)	
	Age	✓	122 (30.3)	
	Lactose intolerance	✓	53 (13.2)	
	I don’t know	-	30 (7.4)	
Months of the year when sunshine is sufficient for vitamin D production	January	×	16 (4.0)	
	February	×	15 (3.7)	
	March	×	43 (10.7)	
	April	✓	150 (37.2)	
	May	✓	279 (69.2)	
	June	✓	365 (90.6)	
	July	✓	377 (93.5)	
	August	✓	352 (87.3)	
	September	✓	184 (45.7)	
	October	×	35 (8.7)	
	November	×	13 (3.2)	
	December	×	12 (3.0)	
Average daily sun exposure required for fair-skinned to get enough vitamin D	Less than 9minutes per day	×	21 (5.2)	
	9–14minutes per day	✓	66 (16.4)	
	15–20minutes per day	×	123 (30.5)	
	More than 20minutes per day	×	86 (21.3)	
	I don’t know	-	107 (26.6)	
Average daily sun exposure required for non-fair-skinned person would need to spend in the sun to get enough vitamin D	Less than 15minutes per day	×	52 (12.9)	
	15–24minutes per day	✓	67 (16.6)	
	About 25–38minutes per day	×	76 (18.9)	
	More than 38minutes per day	×	74 (18.4)	
	I don’t know	-	133 (33.0)	
	Missing	×	1 (0.2)	
Recommended daily dose	Value entered was below recommended	×	10	2.3
	Correct dose entered	✓	61	15.1
	Value entered was above recommended	×	33	13.3

Ticks ✓denote the correct answers and crosses × indicate an incorrect answer.

### Vitamin D supplementation practices

Vitamin D supplements were taken by 64.3% of participants, of which 37.5% took a vitamin D supplement every day and 25.8% less frequently (Table 3).

**Table 3.** Average frequency participants took a vitamin D supplement during the winter (n = 403).

Frequency	N	Percent
Every day	151	37.5
Several times a week	57	14.1
Once a week	20	5.0
Every couple of weeks	22	5.5
Monthly	9	2.2
Never	144	35.7

Of those who took vitamin D supplements, the recommended dose of 10 µg/400IU was taken daily by 22.8% (n = 30) participants (Table 4). The majority of vitamin D supplement takers took more than 10 µg/400IU per day (62.9%). A few participants took less than 10 µg/400IU per day (5.3%), with 9.1% unsure of the dose taken. Within the whole sample, 7.4% of participants took a 10 µg/400IU vitamin D supplement daily during the autumn/winter months.

**Table 4.** Doses of vitamin D taken daily (n = 132). Ticks ✓denote the correct answers, crosses × indicate an incorrect answer.

Units	Dose	✓/ ×	N	Percent of respondents
Micrograms	Below recommended 10µg	×	6	4.5
	10 µg	✓	29	22.0
	Above recommended 10 µg	×	69	52.3
IUs	Below recommended 400 IUs	×	1	0.8
	400 IUs	✓	1	0.8
	Above recommended 400 IUs	×	14	10.6
	Don’t know	-	12	9.1

Participants who scored above average for vitamin D knowledge were statistically more likely to take vitamin D supplements daily compared to participants with below average knowledge scores who were less likely to supplement daily (OR = 1.9, 1.5–2.3), *X*^2^ (1, *N* = 401) = 6.7, p = 0.009 and at the correct dose *X*^2^ (1, *N* = 120) = 4.1, p = 0.043.

There was a statistically significant association between age and frequency of vitamin D supplementation (stratified into daily supplementation and less frequently). Those who were aged 18–24 and aged ≥65 years were less likely to take a vitamin D supplement daily, and those who were aged 25–64 years were more likely to take a vitamin D supplement every day, *X*^2^ (4, *N* = 402) = 44.6, p < 0.001. There was no association between age and taking the recommended dose of vitamin D supplements (p > 0.05).

When stratified into ‘white Scottish’, ‘non-white not Scottish’ and ‘white not Scottish’, participants who were white Scottish were more likely to take the recommended dose of vitamin D, *X*^2^ (2, *N* = 118) = 28.5, p < 0.001. However, the frequency of dosages was not statistically significant (p > 0.05).

There were no associations between other demographic factors, gender, education, SIMD, employment or income, with frequency of vitamin D supplementation or supplementing the recommended dose of vitamin D (p > 0.05).

For those who did not take a vitamin D supplement daily during autumn/winter (n = 252), participants were advised of the current guidance in the survey, of which 76.6% considered themselves as either ‘likely’ or ‘very likely’ to start adhering to the recommendation.

### Predicting vitamin D supplement use

Supplement use logistical regression results are summarised in Table 5. There were no factors identified to predict daily vitamin D supplement use (p > 0.05).

**Table 5.** Factors contributing to vitamin D supplementation use.

							95% CI for OR	
	B	SE	Wald	df	p	OR	Lower	Upper
Knowledge score	−0.36	0.11	10.62	1	0.09	0.70	0.57	0.87
Gender Males	−0.75	0.99	0.56	1	0.45	0.47	0.07	3.33
Gender Females	−0.38	−0.29	1.77	1	0.19	0.69	0.39	1.20
Educated to degree level or above	0.09	0.25	0.13	1	0.72	1.10	0.67	1.78
Age	−0.05	0.01	34.90	1	0.06	0.95	0.93	0.97
SIMD 1	−0.76	0.46	2.80	1	0.09	0.47	0.19	1.14
SIMD 2	−0.55	0.46	1.41	1	0.24	0.58	0.23	1.43
SIMD 3	−0.42	0.45	0.87	1	0.35	0.66	0.28	1.56
SIMD 4	−0.22	0.44	0.26	1	0.61	0.80	0.39	1.90
SIMD 5								
Constant	4.15	0.65	40.71	1	0.073	63.43		

Logistic regression predicting likelihood of supplement use on relevant factors (n = 393, n = 11 missing data).

B: B coefficient; CI: confidence interval; df: degrees of freedom; OR: odds ratio; p: p value; SE: standard error of mean; SIMD: Scottish Index of Multiple Deprivation; Wald: Wald chi-square test.

### Vitamin D supplement purchasing

In terms of where vitamin D supplements were purchased, the majority were from supermarkets (37.1%) and chemists/pharmacies (24.9%). Most participants took vitamin D supplements in tablet form (Table 6). There was a wide variation in vitamin D supplement brands purchased, with high street stores being most frequent. The median (25th and 75th) spend on vitamin D supplements was £3.00 (1.00, 5.00) per month (n = 128).

**Table 6.** Vitamin D supplement purchase location (n = 205) and form (n = 163).

Question	Response	N	Percent
Place of purchase	Supermarket	76	37.1
	Health food store	37	18.0
	Chemist/Pharmacy	51	24.9
	Online	32	15.6
	Other	9	4.4
Form of supplement	Tablets	119	73.0
	Caplets	18	11.0
	Spray	7	4.3
	Drops	3	1.8
	Jellies	11	6.7
	Chewable tablets	5	3.1

### Barriers to vitamin D supplementation

The most frequent reasons as to why daily vitamin D supplementation was not followed during autumn/winter months were forgetfulness (30.1%) and lack of awareness of the recommendations (22.1%). Twelve percent of individuals preferred to get vitamins from food and 8% did not see the need for taking vitamin D. Of those who answered ‘other’, responses ranged from medical conditions that contraindicated vitamin D supplementation advice, to beliefs that sufficient vitamin D could be obtained from food or sunlight (Table 7).

**Table 7.** Barriers to following the recommended daily vitamin D supplement advice (n = 362).

Reasons	N	Percentage of responses
I'm unaware of the recommended advice	80	22.1
I'm confused by the recommended advice	20	5.5
I don't see the need	32	8.8
I forget	109	30.1
I prefer to get vitamins from food	44	12.2
I can't afford supplements	13	3.6
I don't know where to buy them	6	1.7
I don't like taking supplements	26	7.2
I don’t agree with taking tablets	5	1.4
I find it hard to swallow tablets	8	2.2
Other	19	5.2

## Discussion

This is the first study to quantitatively investigate the knowledge of vitamin D, practices of vitamin D supplementation and identify barriers to vitamin D supplement use in adults living in Scotland. The results suggest that overall knowledge scores of vitamin D are low (31.4%) in the Scottish population, however identification of vitamin D's role in bone health (80.6%) and immunity (70.2%) along with sources of vitamin D, including sun (83.9%) and vitamin D supplements (81.9%), were good. There was poorer knowledge of dietary sources of vitamin D, which is concerning considering that only 37.5% of the individuals in the current study reported to take vitamin D supplements daily during autumn and winter months.

## Vitamin D knowledge

In the current study awareness of vitamin D was good at 99.5%, which corroborates the 99% reported in the UK-based study by O’Connor et al. (2018). However, knowledge of vitamin D was poor with an average score of only 31.4%, another similar trend reported in the literature (Kung and Lee, 2006; Boland et al., 2015). This result was not dissimilar to the findings by Boland et al. (2015) where the mean score was 29% in an adult Canadian population.

For identifying sources of vitamin D the participants in the current study outperformed the respondents in research conducted by Kung and Lee (2006) and Toher et al. (2014), particularly in recognition of sunlight (83.9% vs 52.6–74.0%) and supplements (81.9% vs 10.0–38.4%). Source knowledge results were more aligned with the reported findings of O’Connor et al. (2018) and Vu et al. (2010), although overall knowledge remains lower than that seen in the published findings by O’Connor et al. (2018) where the mean score was 56.6%. However, it should be mentioned that the higher knowledge score reported by O’Connor et al. (2018) is likely to have been influenced by the high level of nutrition students who participated in their research. Lack of knowledge of vitamin D, including dietary sources and government recommendations, was apparent in published findings by Kotta et al. (2015), who conducted qualitative interviews with adults living in England. However, knowledge was not quantitatively scored in the aforementioned study. In the current study, there was a high proportion of females (72.7%) who have been reported elsewhere as having better knowledge of vitamin D (Kung and Lee, 2006; Boland et al., 2015). Miller and Russell (2004) report that American females of childbearing age have good knowledge of dietary supplements, however there was no difference between gender and knowledge of vitamin D in the current study.

## Vitamin D supplements

In terms of understanding the vitamin D guidance, only 130 participants attempted to identify the recommended daily dose in the current study, of which 61 identified the recommended dose, which represents only 15.1% of the total participants. Although this is low, it is an improvement on the 5% of participants who correctly identified the reference nutrient intake (RNI) of vitamin D in the FSS survey (Food Standards Scotland, 2020).

Only 7.4% of participants in the current study took the recommended dose of vitamin D daily during autumn-winter months (10 µg/400IUs). This is a slight improvement on the 5% seen by FSS (2020), which may indicate the FSS campaign had a minor impact. Almost 20% reported consuming doses of vitamin D above the RNI which could raise some concerns about potential toxicity for those with medical conditions, however health status was not monitored in the current work and therefore doses of up to 100 µg are deemed tolerable as per general population advice. The evidence on vitamin D toxicity tends to be based on low-quality case studies, which can be useful to identify risk factors for over-supplementation (Agbabiaka et al., 2018) and potential side effects (De Vincentis et al., 2021). However, they do not offer robust evidence of the impact of over-supplementation, which might lead to nothing more than abnormal blood results (Kilbane et al., 2014). Reported symptoms may potentially be due to co-administration of calcium leading to hypercalcemia, rather than from vitamin D alone (European Food Safety Authority, 2016).

A potential source of confusion leading to under-supplementation, seen in around 5% of those who took <10 µg daily vitamin D in the current study, may be inconsistencies in RNIs on supplement labels. Some supplement labels state the RNI for vitamin D as 5 µg per day as food labelling is standardised at EU level. Products sold in the UK are required to be labelled in accordance with the EU based RNI for labelling, which is 5 µg (Food Standards Scotland, 2022b). Recommending a daily supplement year-round in Scotland may improve adherence by simplifying the regime (Vlasnik et al., 2005) as 30.1% of the adults included in the current work report that forgetfulness was a common barrier to following the vitamin D supplement recommendation. It seems reasonable to suggest remembering to restart taking vitamin D supplements in autumn-winter may also be an issue. Daily use would also avoid issues with factors that decrease absorption from sunlight and may give better population confidence by removing ambiguity. In addition, supplementation may also be a more advantageous method to optimise vitamin D levels given that only 29% and 33% of Scottish men and women, respectively, are reported to consume oily fish once a week or more, a key source of dietary vitamin D (Scottish Health Survey, 2020).    

The median spend on vitamin D supplements was £3.00 per month, which can be considered a reasonable cost given that 45.6% of the participants in the current study had a household income of £700 per week/£36,400 per annum or above. It is important that vitamin D supplements remain affordable and accessible to the public, especially at present with food and energy price inflation. Signposting individuals to locations where safe and low-priced vitamin D supplements can be purchased would be advantageous, particularly those on low incomes who are not eligible for the free Scottish Vitamins Scheme or Healthy Start initiative (pregnant and breastfeeding females and children under 3 only). This could be a consideration for future vitamin D campaigns, educating the public not only on the health benefits of vitamin D but also where low-cost vitamin D supplements can be found. Vitamin D supplements from supermarkets, particularly own brand products, could be recommended. Considering that brand familiarity can impact purchase intention (Castro et al., 2018), consumers may be more likely to buy branded vitamin supplements than supermarket own-brand products due to consumer belief that branded supplements are of higher quality and more effective than supermarket own-brand products.

## Public health campaigns

Only 8.2% of participants in this study recalled the FSS vitamin D campaign (2021–2022). It can therefore be suggested that awareness of the recommendations has more likely come from other sources, namely educational environments (60.5% educated to degree level or above), family and health professionals, as reported in our findings. The current study had a demographic with >10.9% participants resident in Scotland for less than a year, which means they may not have been resident in Scotland for the entire duration of the FSS campaign.

In terms of where the campaign was viewed, social media was the most common avenue with just over half of those who recalled the campaign naming it as a source, with TV and radio significantly lower at 27.0% and 21.0%, respectively. This may indicate declining impact from traditional forms of media to gain health information (Stellefson et al., 2020). Although, this could have been confounded by the relatively young cohort mean age 36.4 years, with younger adults more likely to use social media (Khoros, 2022). However, knowledge alone does not guarantee behaviour change (Baker and Murdoch 2008) and some interventions can unintentionally have negative impacts on behaviours (Nyhan et al., 2014; Stead et al., 2019). There is a need for research on the impact of ‘new media’ (e.g. liking, sharing or commenting on content and downloading campaign apps) and the cost effectiveness of messages related to health, in order to improve public health interventions (Stead et al., 2019).

From a theoretical point of view, the Health Belief Model (Becker, 1974) and Expanded Health Belief Model (Strecher and Rosenstock, 1997) assume that people will perform a health-promoting behaviour (such as vitamin intake) if they perceive a serious health threat, believe that the health behaviour will reduce the health threat, perceive minimal barriers and believe they can effectively execute the health behaviour. Accordingly, if Scottish adults are aware of the importance of taking vitamin D to support health but do not know where to get vitamin D other than from the sun or dietary supplements (a barrier that reduces feelings of self-efficacy) they are less likely to seek out these sources and engage with the desired health behaviour. Perhaps the noted lack of vitamin D knowledge from dietary sources, number of individuals who do not take daily vitamin D supplements (over a third of the current cohort never take vitamin D supplements) and high northern latitude of Scotland combined may contribute to the prevalence of vitamin D deficiency in Scotland. However, we acknowledge the need for updated deficiency data, with the most recent vitamin D deficiency data based on Scottish Health Survey data from 2010 to 2011. Ten micrograms of vitamin D is considered a safe daily dose for the general population (SACN, 2016) and given that there is evidence to suggest that Scottish individuals may benefit from daily vitamin D supplementation all year round (Zgaga et al., 2011) we suggest that public health guidance should consider updating advice for Scottish adults to supplement with 10 µg daily, not only during autumn-winter months and not only for high-risk groups.

## Limitations and future research

As with all cross-sectional studies it is not possible to comment on the direction of associations. As the majority of the data was quantitative it is difficult to interpret why people are not currently taking vitamin D, aside from the limited number of options offered in the survey. Hard copies of the questionnaire should have been utilised to reach older adults who may be less likely to use the internet, therefore future research should allow the option for electronic and hard copy questionnaires to be used.

The use of convenience sampling is likely to have introduced a degree of bias; this approach meant that individuals associated with a Higher Education institution were predominantly recruited (staff and students) and this is likely to have led to a skewed dataset towards a higher level of education and younger age group, impacting on the generalisability of the results. In terms of age it should be noted that 29% of our study population were <25 and just 4% were >65 compared to 12% and 21%, respectively, reported by FSS. At risk population groups were underrepresented in the current work, and hence studies focussing on pregnant/breastfeeding females, children, older adults >65years and individuals with darker skin are warranted. This is important as recent evidence from Tanna et al. (2023) report that increasing age is negatively associated with vitamin D supplement intake in British adults. Although the current study did have a larger sample included than other similar work (O’Connor et al. 2018; Toher et al., 2014), a larger sample would enable better understanding of the diverse Scottish population.

Data were stratified using mean and medians, therefore it is important to acknowledge the drawbacks of taking such approach, including sensitivity to outliers, loss of precision and potential impact on statistical testing, that is, when assumptions of normal distribution are required (Field, 2017). Scoring of the questionnaire followed the protocol adopted by Boland et al. (2015) which allowed for direct comparison of scores with the current data, however this scoring technique was perhaps cumbersome with some correct answers weighted for as little as 1/7 or 1/13 of a mark.

Logistic regression was performed to determine whether age, gender, SIMD or knowledge were predictors of daily vitamin D supplementation, which were not shown to be significant in the current study. This may have been contributed to by the demographics of the group being skewed towards a younger, more educated group and the limitations of SIMD as an indicator of relative affluence, particularly in a student population. This may be an avenue for further research.

A further limitation of this study was that some of the deviation in reported dosing regimens for vitamin D may have been due to individualised medical advice. Around 38% of respondents reported hearing about vitamin D from a doctor or nurse, and although they may have received generic advice around the public health guidance (10 µg daily) it is also possible that they were given specific regimens due to certain health conditions that would have required either high loading doses followed by maintenance, or reduced doses due to concerns of toxicity. It would be important in any future research to clearly identify cases with medical conditions that may require altered dosing regimes as per national guidance (NICE, 2022). Furthermore, during the COVID pandemic residents in Scotland, as well as other parts of the UK, were provided free vitamin D 10 µg supplements over the wintertime which may have affected respondent awareness. It is reported that 40% of Scottish shielders took up the offer of free vitamin D (Scottish Government, 2021). The population studied had very good knowledge of vitamin D's role in immunity, the prevention of rickets and bone health yet over 63% of the sample who took daily vitamin D supplements during autumn-winter took more than the recommended dose to support skeletal on bone health (>10 µg). This highlights a future avenue for exploration with regard to understanding knowledge and practices of at-risk individuals with impaired immune function and rickets.

## Conclusion

In conclusion, the current study highlights the need to improve both knowledge of vitamin D and practice of vitamin D supplementation during the autumn and winter months in Scotland. It is clear that despite the effort of FSS to raise awareness of vitamin D, there is still a significant gap in the Scottish population's knowledge of vitamin D and improvements need to be made to supplementation practices. The true extent to which poor supplementation practices translate into vitamin D deficiency is currently unknown, especially since deficiency data in Scottish adults has not been updated in over a decade.

## Supplemental Material

sj-docx-1-nah-10.1177_02601060241238824 - Supplemental material for Knowledge of vitamin D and practices of vitamin D supplementation in a Scottish adult population: A cross-sectional study Supplemental material, sj-docx-1-nah-10.1177_02601060241238824 for Knowledge of vitamin D and practices of vitamin D supplementation in a Scottish adult population: A cross-sectional study by Suzanne M.M. Zaremba and Karen Conduit-Turner in Nutrition and Health

sj-docx-2-nah-10.1177_02601060241238824 - Supplemental material for Knowledge of vitamin D and practices of vitamin D supplementation in a Scottish adult population: A cross-sectional study Supplemental material, sj-docx-2-nah-10.1177_02601060241238824 for Knowledge of vitamin D and practices of vitamin D supplementation in a Scottish adult population: A cross-sectional study by Suzanne M.M. Zaremba and Karen Conduit-Turner in Nutrition and Health

## Appendix

### Demographics

**1. Age** in years ___________________
**2. Gender**:
  □ Male
  □ Female
  □ Other (with a blank entry field for the participant to self-identify)
  □ Prefer not to answer
**3. Are you currently pregnant or breastfeeding?**
  □ Yes
  □ No
  □ Prefer not to answer
**4. Employment:**
  □ F/T employment
  □ P/T employment
  □ Not currently employed
  □ Student
  □ Retired

**Household Income:**

**5. What was your household's total income from all sources over the last 12 months*** **:
  *Count income from every person in the household including benefits.
  **Do not deduct tax/National Insurance/Health insurance payments/Superannuation
  □ Less than £100 per week OR less than £5200 per year
  □ £100–199 per week or £5200 to £10,399 per year
  □ £200–299 per week or £10,400 to £15,499 per year
  □ £300–399 per week or £15,500 to £20,799 per year
  □ £400–499 per week or £20,800 to £25,999 per year
  □ £500–699 per week or £26,000 to £36,399 per year
  □ £700–999 per week or £36,400 to £51,999 per year
  □ £1000–1499 per week or £52,000 to £77,999 per year
  □ £1500 per week or more to £78,000 or more per year
  □ Prefer not to answer
**6. Highest level of education**
  □ O Grade, Standard Grade, National 3, 4 or 5, Intermediate 1 or 2, GCSE, CSE or equivalent
  □ Higher, Advanced Higher, SCE Higher Grade, CSYS, A Level, AS Level or equivalent
  □ Apprenticeship (trade or equivalent)
  □ Apprenticeship (Foundation or equivalent)
  □ Apprenticeship (Modern or equivalent)
  □ Apprenticeship (Graduate or equivalent)
  □ GSVQ Foundation or Intermediate, SVQ level 1 or 2, SCOTVEC Module, City and Guilds Craft or equivalent
  □ GSVQ Advanced, SVQ level 3, ONC, OND, SCOTVEC National Diploma, City and Guilds Advanced Craft or equivalent
  □ HNC, HND, SVQ level 4 or equivalent
  □ Other school qualifications not already mentioned (including foreign qualifications)
  □ Other post-school but pre-Higher Education qualifications not already mentioned (including foreign qualifications)
  □ Degree, Postgraduate qualifications, Masters, PhD, SVQ level 5 or equivalent
  □ Professional qualifications (for example, teaching, nursing, accountancy) Other Higher Education qualifications not already mentioned (including foreign qualifications)
  □ No qualifications
  □ Prefer not to answer

**Nationality**

7. What is your nationality? **___________________**

**Residence**

**8. How long have you lived in Scotland**: _______ years __________months
**9. Postcode** ____________ (Please include full postcode e.g. DD1 1HG)

**Ethnicity:**

**10. How would you describe your ethnic origin? (Tick one box only)**

What is your ethnic group? Choose ONE section from A to F, then tick ONE box which best describes your ethnic group or background:

A White:

□ Scottish
□ Other British
□ Irish
□ Polish
□ Gypsy / Traveller
□ Roma
□ Showman / Showwoman
Other white ethnic group, please write in: _________

B Mixed or multiple ethnic groups:

□ Any mixed or multiple ethnic groups, please write in: __________

C Asian:

□ Scottish Asian or British Asian Pakistani, Scottish Pakistani or British

Pakistani:

□ Indian, Scottish Indian or British Indian
□ Bangladeshi, Scottish Bangladeshi or British Bangladeshi
□ Chinese, Scottish Chinese or British Chinese
□ Other, please write in: __________

D □ African, Scottish African or British African:

Please write in (for example, NIGERIAN, SOMALI): __________

E □ Caribbean or Black:

Please write in (for example, SCOTTISH CARIBBEAN, BLACK SCOTTISH): ___________

F □ Other ethnic group

Arab, Scottish Arab or British Arab Other, please write in (for example, SIKH, JEWISH) ___________

### Vitamin D awareness

**11. Have you heard of vitamin D?**
  □ Yes
  □ No

If NO reroute to Information-point 16

**12. Where did you hear about vitamin D (tick all that apply)?**
  □ Doctor/ Nurse
  □ Other Health Professional
  □ School / college / university
  □ Newspaper
  □ Family
  □ Friend
  □ Television
  □ Radio
  □ Poster/ Billboard
  □ Magazine
  □ Book
  □ Social media
  □ I don’t know
  □ Other______

### Knowledge of vitamin D

**13. Vitamin D helps with which of the following health effects?** (Check all that apply)
  □ Bone health
  □ Immune health
  □ Prevention of Rickets
  □ Vision Health
  □ Hair Growth
  □ Prevention of diabetes
  □ Cardio-vascular health
  □ Cognitive health
  □ Cancer prevention
  □ Skin softness
  □ Calcium absorption
  □ None of the above
  □ Other_______
  □ I don’t know
**14. Where do you think vitamin D comes from?** (Check all that are correct)
  □ Fruits
  □ Vegetables
  □ Fatty fish
  □ Vitamin D supplements
  □ Select cereals
  □ Milk/ Dairy
  □ Nuts
  □ Cod liver
  □ Sun
  □ Eggs
  □ Mushrooms
  □ I don’t know
**15. Factors that can decrease the amount of vitamin D a person can get are (check all that apply):**

**Table:**

□ Skin pigment	□ Pregnancy/ lactation	□ Dairy allergy
□ Shade/ clouds	□ Fatty diets	□ Pollution
□ Time of day	□ Sunscreen use	□ Wind
□ Latitude	□ Vegan diet	□ Smoking
□ Season	□ Vegetarian diets	□ I don’t know
□ Age	□ Lactose intolerance	

**16. Information page with no questions will be displayed on JISC with the following text:**

Although some foods can be a source of vitamin D it is incredibly difficult to get enough from food alone. The most effective sources, other than from vitamin D supplements is the sun.

**17. How much time would the average fair-skinned person need to spend in the sun to get enough vitamin D, if their bare legs and arms were exposed and without sunscreen?**
  □ Less than 9minutes per day
  □ 9–14minutes per day
  □ 15–20minutes per day
  □ More than 20minutes per day
  □ I don’t know
**18. How much time would the average non-fair-skinned (i.e. non-Caucasian) person need to spend in the sun to get enough vitamin D, if their bare legs and arms were exposed?**
  □ Less than 15minutes per day
  □ 15–24 minutes per day
  □ About 25–38minutes per day
  □ More than 38minutes per day
  □ I don’t know
**19. In Scotland, during which months of the year is the sunshine adequate to enable adults to make vitamin D, if their bare legs and arms were exposed? (Tick all months that apply)**
  □ January
  □ February
  □ March
  □ April
  □ May
  □ June
  □ July
  □ August
  □ September
  □ October
  □ November
  □ December
**20. Are you aware of the recommendation for adults to consider taking a daily vitamin D supplement between October and March each year in Scotland?**
  □ Yes
  □ No
**21. How many micrograms of vitamin D should you take as a daily supplement living in Scotland?**

_______ micrograms

□ Don't know

### Vitamin D practices

**22. On average how often, if at all, do you take a vitamin D supplement during the winter months?**
  □ Every day (route to Q23)
  □ Several times a week (route to Q24)
  □ Once a week (route to Q24)
  □ Every couple of weeks (route to Q24)
  □ Monthly (route to Q24)
  □ Never (route to Q24)
**23. What dose of vitamin D do you take in the winter months?** _______ micrograms per day

(to convert international units to micrograms take the IU and divide it by 40. For example: 800 IU vitamin D / 40 = 20 micrograms vitamin D) (route to Q27)

**24. If you don’t take a supplement every day, what is the reason? Please select all that apply**
  □ I'm unaware of the recommended advice
  □ I'm confused by the recommended advice
  □ I don't see the need
  □ I forget
  □ I prefer to get vitamins from food
  □ I can't afford supplements
  □ I don't know where to buy them
  □ I don't like taking supplements
  □ I don’t agree with taking tablets
  □ I find it hard to swallow tablets
  □ Other, please state (____________)
**25. Information page with no questions will be displayed on JISC with the following text:** In Scotland we only get enough of the right kind of sunlight for our bodies to make vitamin D between April and September, mostly between 11am and 3pm. Some people, including people from minority ethnic groups with darker skin, are at higher risk of vitamin D deficiency and should take a daily supplement all year round. Taking a daily 10 microgram vitamin D supplement, particularly between October and March, supports bone and muscle health and reduces our risk of vitamin D deficiency.
**26. Knowing these benefits now, how likely are you to consider taking a daily 10 microgram vitamin D supplement between October and March?**
  □ Very likely
  □ Likely
  □ Neither likely nor unlikely
  □ Unlikely
  □ Very unlikely

### Supplements

**27. Where do you buy your vitamin D supplements?**
  □ Supermarket
  □ Health food store
  □ Chemist/Pharmacy
  □ Online
  □ Other, please state ____________
**28. What form of vitamin D supplement do you use?**
  □ Tablets
  □ Caplets
  □ Spray
  □ Drops
  □ Jellies
  □ Chewable tablets
**29. Which brand of vitamin D supplement do you currently use?** _____________________
  □ Don’t know the brand
**30. On average, how much do you spend on vitamin D supplements per month?** £_________
  □ Don’t know
**31. Please indicate how many times, on average, you have taken the following supplements during the last 3 months:**

**Table:**

	Never or <1 a month	1–3 a month	Once a week	2–4 a week	5–6 a week	Once a day	2–3 a day	4–5 a day	6 + a day
Multivitamin									
Calcium									
Calcium + Vitamin D									

**FSS Campaign**

**32. Did you see/hear about the Food Standards Scotland campaign about vitamin D over the winter months?**
  □ Yes (if yes route to Q33)
  □ No (route to point 34)
  □ Can’t remember (route to point 34)

**33. Where­­­­­­­­­­­­ did you see/hear about the FSS campaign (please tick all that apply)**
  □ Newspaper
  □ Television
  □ Radio
  □ Poster/ Billboard
  □ Magazine
  □ Social media
  □ Other______
**34. End of survey. Thank you for taking the time to complete this survey**
