# Supplementary material for: Knowledge of vitamin D and practices of vitamin D supplementation in a Scottish adult population: A cross-sectional study
Source: Nutr Health. 2024 Mar 18;31(2):715–28. doi: 10.1177/02601060241238824 (PMC12174623; doi:10.1177/02601060241238824)
Supplement: sj-docx-1-nah-10.1177_02601060241238824 - Supplemental material for Knowledge of vitamin D and practices of vitamin D supplementation in a Scottish adult population: A cross-sectional study [file sj-docx-1-nah-10.1177_02601060241238824.docx]

**Demographics**

**1. Age** in years ___________________

**2. Gender**:

□ Male

□ Female

□ Other (with a blank entry field for the participant to self-identify)

□ Prefer not to answer

**3. Are you currently pregnant or breastfeeding?**

□ Yes

□ No

□ Prefer not to answer

**4. Employment:**

□ F/T employment

□ P/T employment

□ Not currently employed

□ Student

□ Retired

**Household Income**:

**5. What was your household’s total income from all sources over the last 12 months*** **:

*Count income from every person in the household including benefits.

**Do not deduct tax/National Insurance/Health insurance payments/Superannuation

□ Less than £100 per week OR Less than £5200 per year

□ £100-199 per week or £5200 to £10,399 per year

□ £200-299 per week or £10,400 to £15,499 per year

□ £300-399 per week or £15,500 to £20,799 per year

□ £400-499 per week or £20,800 to £25,999 per year

□ £500-699 per week or £26,000 to £36,399 per year

□ £700-999 per week or £36,400 to £51,999 per year

□ £1000-1499 per week or £52,000 to £77,999 per year

□ £1500 per week or more to £78,000 or more per year

□ Prefer not to answer

**6. Highest level of education**

□ O Grade, Standard Grade, National 3, 4 or 5, Intermediate 1 or 2, GCSE, CSE or equivalent

□ Higher, Advanced Higher, SCE Higher Grade, CSYS, A Level, AS Level or equivalent

□ Apprenticeship (trade or equivalent)

□ Apprenticeship (Foundation or equivalent)

□ Apprenticeship (Modern or equivalent)

□ Apprenticeship (Graduate or equivalent)

□ GSVQ Foundation or Intermediate, SVQ level 1 or 2, SCOTVEC Module, City and Guilds Craft or equivalent

□ GSVQ Advanced, SVQ level 3, ONC, OND, SCOTVEC National Diploma, City and Guilds Advanced Craft or equivalent

□ HNC, HND, SVQ level 4 or equivalent

□ Other school qualifications not already mentioned (including foreign qualifications)

□ Other post-school but pre-Higher Education qualifications not already mentioned (including foreign qualifications)

□ Degree, Postgraduate qualifications, Masters, PhD, SVQ level 5 or equivalent

□ Professional qualifications (for example, teaching, nursing, accountancy) Other Higher Education qualifications not already mentioned (including foreign qualifications)

□ No qualifications

□ Prefer not to answer

**Nationality**

7. What is your nationality? **___________________**

**Residence**

**8. How long have you lived in Scotland**: __________________ years __________months

**9. Postcode** ________________________ (Please include full postcode e.g. DD1 1HG)

**Ethnicity**:

**10. How would you describe your ethnic origin? (Tick one box only)**

What is your ethnic group? Choose ONE section from A to F, then tick ONE box which best describes your ethnic group or background:

A White:

□ Scottish

□ Other British

□ Irish

□ Polish

□ Gypsy / Traveller

□ Roma

□ Showman / Showwoman

Other white ethnic group, please write in: _________

B Mixed or multiple ethnic groups:

□ Any mixed or multiple ethnic groups, please write in: __________

C Asian:

□ Scottish Asian or British Asian Pakistani,Scottish Pakistani or British

Pakistani:

□ Indian, Scottish Indian or British Indian

□ Bangladeshi, Scottish Bangladeshi or British Bangladeshi

□ Chinese, Scottish Chinese or British Chinese

□ Other, please write in: __________

D □ African, Scottish African or British African:

Please write in (for example, NIGERIAN, SOMALI): __________

E □ Caribbean or Black:

Please write in (for example, SCOTTISH CARIBBEAN, BLACK SCOTTISH): _____________

F □ Other ethnic group

Arab, Scottish Arab or British Arab Other, please write in (for example, SIKH, JEWISH) ____________

**Vitamin D Awareness**

**11. Have you heard of vitamin D?**

□ Yes

□ No

If NO reroute to Information-point 16

**12.** **Where did you hear about vitamin D (tick all that apply)?**

□ Doctor/ Nurse

□ Other Health Professional

□ School / college / university

□ Newspaper

□ Family

□ Friend

□ Television

□ Radio

□ Poster/ Billboard

□ Magazine

□ Book

□ Social media

□ I don’t know

□ Other______

**Knowledge of vitamin D**

**13. Vitamin D helps with which of the following health effects?** (check all that apply)

□ Bone health

□ Immune health

□ Prevention of Rickets

□ Vision Health

□ Hair Growth

□ Prevention of diabetes

□ Cardio-vascular health

□ Cognitive health

□ Cancer prevention

□ Skin softness

□ Calcium absorption

□ None of the above

□ Other_______

□ I don’t know

**14. Where do you think vitamin D comes from?** (check all that are correct)

□ Fruits

□ Vegetables

□ Fatty fish

□ Vitamin D supplements

□ Select cereals

□ Milk/ Dairy

□ Nuts

□ Cod liver

□ Sun

□ Eggs

□ Mushrooms

□ I don’t know

**15. Factors that can decrease the amount of vitamin D a person can get are (check all that apply):**

| □ Skin pigment | □ Pregnancy/ lactation | □ Dairy allergy |
| --- | --- | --- |
| □ Shade/ clouds | □ Fatty diets | □ Pollution |
| □ Time of day | □ Sunscreen use | □ Wind |
| □ Latitude | □ Vegan diet | □ Smoking |
| □ Season | □ Vegetarian diets | □ I don’t know |
| □ Age | □ Lactose intolerance |  |

**16. Information page with no questions will be displayed on JISC with the following text:**

Although some foods can be a source of Vitamin D it is incredibly difficult to get enough from food alone. The most effective sources, other than from Vitamin D supplements is the sun.

**17. How much time would the average fair-skinned person need to spend in the sun to get enough vitamin D, if their bare legs and arms were exposed and without sunscreen?**

□ Less than 9 minutes per day

□ 9-14 minutes per day

□ 15-20 minutes per day

□ More than 20 minutes per day

□ I don’t know

**18. How much time would the average non-fair-skinned (i.e., non-Caucasian) person need to spend in the sun to get enough vitamin D, if their bare legs and arms were exposed?**

□ Less than 15 minutes per day

□ 15-24 minutes per day

□ About 25-38 minutes per day

□ More than 38 minutes per day

□ I don’t know

**19. In Scotland, during which months of the year is the sunshine adequate to enable adults to make Vitamin D, if their bare legs and arms were exposed? (tick all months that apply)**

□ January

□ February

□ March

□ April

□ May

□ June

□ July

□ August

□ September

□ October

□ November

□ December

**20. Are you aware of the recommendation for adults to consider taking a daily vitamin D supplement between October and March each year in Scotland?**

□ Yes

□ No

**21.How many micrograms of vitamin D should you take as a daily supplement living in Scotland?**

_______ micrograms

□ Don't know

**Vitamin D practices**

**22.** **On average how often, if at all, do you take a vitamin D supplement during the winter months?**

□ Every day (route to Q23)

□ Several times a week (route to Q24)

□ Once a week (route to Q24)

□ Every couple of weeks (route to Q24)

□ Monthly (route to Q24)

□ Never (route to Q24)

**23. What dose of vitamin D do you take in the winter months?** _______ micrograms per day

(to convert international units to micrograms take the IU and divide it by 40. E.g: 800 IU vitamin D / 40 = 20 micrograms vitamin D) (route to Q27)

**24. If you don’t take a supplement every day, what is the reason?** **Please select all that apply**

□ I'm unaware of the recommended advice

□ I'm confused by the recommended advice

□ I don't see the need

□ I forget

□ I prefer to get vitamins from food

□ I can't afford supplements

□ I don't know where to buy them

□ I don't like taking supplements

□ I don’t agree with taking tablets

□ I find it hard to swallow tablets

□ Other, please state (____________)

**25.** **Information page with no questions will be displayed on JISC with the following text:**  In Scotland we only get enough of the right kind of sunlight for our bodies to make vitamin D between April and September, mostly between 11am and 3pm. Some people, including people from minority ethnic groups with darker skin, are at higher risk of vitamin D deficiency and should take a daily supplement all year round. Taking a daily 10 microgram vitamin D supplement, particularly between October and March, supports bone and muscle health and reduces our risk of vitamin D deficiency.

**26. Knowing these benefits now, how likely are you to consider taking a daily 10 microgram vitamin D supplement between October and March?**

□ Very likely

□ Likely

□ Neither likely nor unlikely

□ Unlikely

□ Very unlikely

**Supplements**

**27. Where do you buy your vitamin D supplements?**

□ Supermarket

□ Health food store

□ Chemist/Pharmacy

□ Online

□ Other, please state ____________

**28. What form of vitamin D supplement do you use?**

□ Tablets

□ Caplets

□ Spray

□ Drops

□ Jellies

□ Chewable tablets

**29. Which brand of vitamin D supplement do you currently use?** _____________________

□ Don’t know the brand

**30. On average, how much do you spend on Vitamin D supplements per month?** £_________

□ Don’t know

**31. Please indicate how many times, on average, you have taken the following supplements during the last 3 months:**

|  | Never or <1 a month | 1-3 a month | Once a week | 2-4 a week | 5-6 a week | Once a day | 2-3 a day | 4-5 a day | 6+ a day |
| --- | --- | --- | --- | --- | --- | --- | --- | --- | --- |
| Multivitamin |  |  |  |  |  |  |  |  |  |
| Calcium |  |  |  |  |  |  |  |  |  |
| Calcium +Vitamin D |  |  |  |  |  |  |  |  |  |

**FSS Campaign**

**32. Did you see/hear about the Food Standards Scotland campaign about vitamin D over the winter months?**

□ Yes (if yes route to Q33)

□ No (route to point 34)

□ Can’t remember (route to point 34)


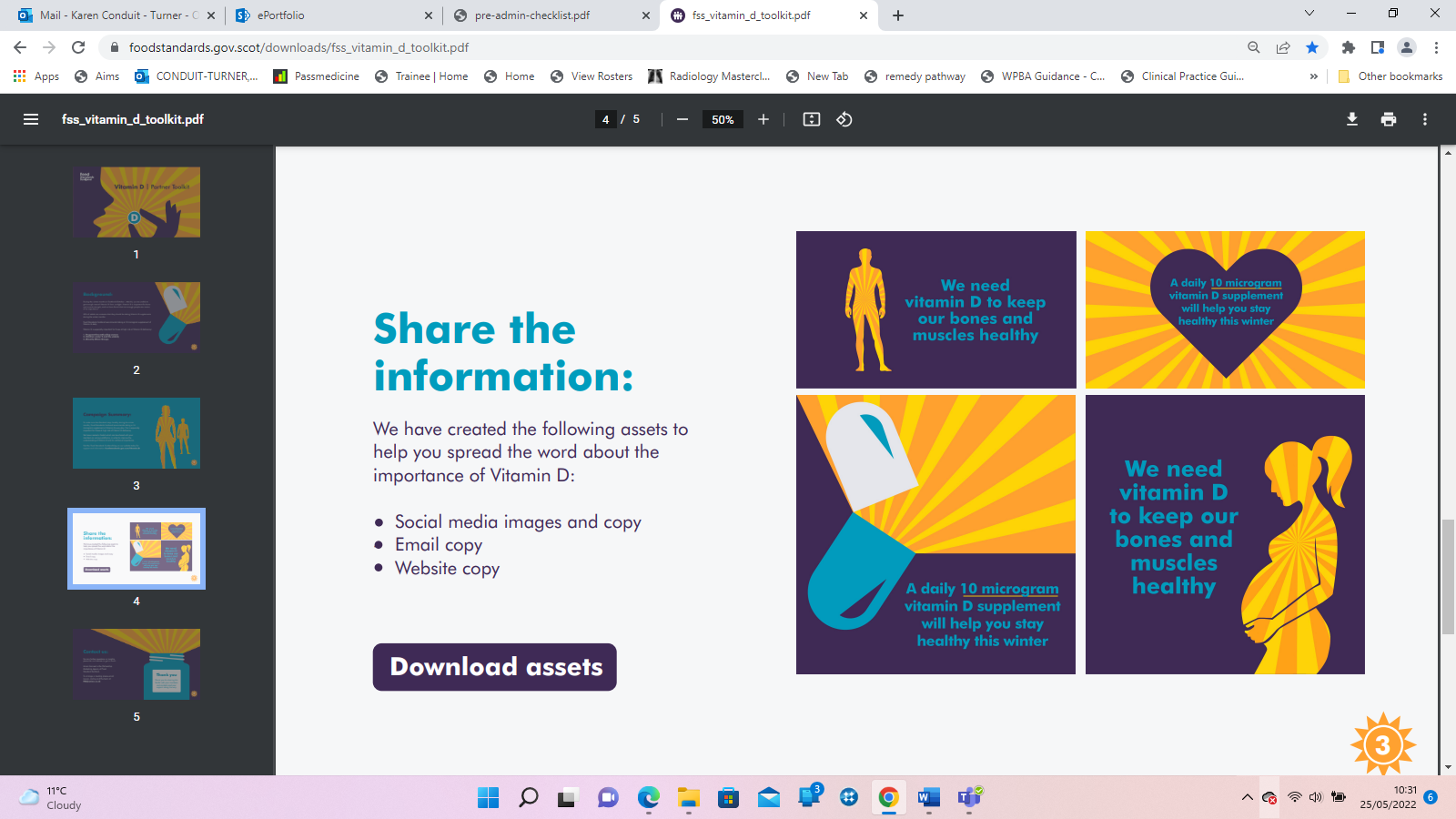


**33. Where­­­­­­­­­­­­ did you see/hear about the FSS campaign (please tick all that apply)**

□ Newspaper

□ Television

□ Radio

□ Poster/ Billboard

□ Magazine

□ Social media

□ Other______

34. End of survey. Thank you for taking the time to complete this survey
